# Nicotinamide Promotes Formation of Retinal Organoids From Human Pluripotent Stem Cells *via* Enhanced Neural Cell Fate Commitment

**DOI:** 10.3389/fncel.2022.878351

**Published:** 2022-06-17

**Authors:** Florian Regent, Zachary Batz, Ryan A. Kelley, Linn Gieser, Anand Swaroop, Holly Y. Chen, Tiansen Li

**Affiliations:** Neurobiology, Neurodegeneration & Repair Laboratory, National Eye Institute, National Institutes of Health, Bethesda, MD, United States

**Keywords:** induced pluripotent stem cells, retinal organoids, nicotinamide, BMP pathway, neuroectoderm

## Abstract

Retinal organoids (ROs) derived from human pluripotent stem cells (hPSCs) recapitulate key features of retinogenesis and provide a promising platform to study retinal development and disease in a human context. Although multiple protocols are currently in use, hPSCs exhibit tremendous variability in differentiation efficiency, with some cell lines consistently yielding few or even no ROs, limiting their utility in research. We report here that early nicotinamide (NAM) treatment significantly improves RO yield across 8 hPSC lines from different donors, including some that would otherwise fail to generate a meaningful number of ROs. NAM treatment promotes neural commitment of hPSCs at the expense of non-neural ectodermal cell fate, which in turn increases eye field progenitor generation. Further analysis suggests that this effect is partially mediated through inhibition of BMP signaling. Our data encourage a broader use of human ROs for disease modeling applications that require the use of multiple patient-specific cell lines.

## Introduction

Retinal organoids (ROs) derived from human pluripotent stem cells (hPSCs) mimic key features of human retinal development and form a laminated structure with time-dependent biogenesis of all major retinal cell types, including photoreceptors with rudimentary outer segment-like structures and partial functionality (Meyer et al., 2011; Nakano et al., 2012; Reichman et al., 2014; Zhong et al., 2014; Kaya et al., 2019; Cowan et al., 2020). Over the past few years, ROs derived from patient induced pluripotent stem cells (hiPSCs) displaying disease-associated phenotypes have provided encouraging proof-of-concept evidence for various treatment approaches, suggesting their potential for retinal degenerative disease modeling and translational medicine (Kaewkhaw et al., 2016; Shimada et al., 2017; Deng et al., 2018; Morizur et al., 2020).

Despite these initial successes and the numerous retinal differentiation protocols now available, limited yield and variability in differentiation efficiency across hPSC lines, likely due to genetic and/or epigenetic variations, are some of the remaining obstacles to a broader adoption of this *in vitro* system (Chichagova et al., 2020; Cowan et al., 2020). Multiple approaches have been developed to overcome these hurdles (Capowski et al., 2019; Mellough et al., 2019) and we recently reported a simplified scraping method for more efficient and convenient production of ROs (Regent et al., 2020). Despite these efforts, we note that some hPSC lines with clinical relevance still show limited yield under multiple differentiation protocols, thus hampering their use in research applications (Chichagova et al., 2020; Cowan et al., 2020).

*In vivo* retinal development begins with the specification of the eye field in the anterior neural plate. These retinal progenitors then pass through distinct states of competence during optic vesicle (OVs) formation and invagination to give rise to retinal pigment epithelium (RPE) and all the major cell types that make up the neural retina (NR). Stem cells cultured *in vitro* can be guided to follow a similar path. While many cell lines proceed through the process efficiently with current protocols, others show much lower yield for reasons that remain unclear. Therefore, identification of extrinsic factors that facilitate and increase the consistency of RO differentiation is still required. One such candidate is nicotinamide (NAM), the amide form of vitamin B3, which has been reported to favor neural and RPE differentiation from hPSC and display anti-apoptotic functions on hPSC-derived neural cells (Cimadamore et al., 2009; Idelson et al., 2009; Buchholz et al., 2013; Griffin et al., 2013, 2017; Meng et al., 2018; Regent et al., 2019, 2020). As both RPE and NR emerge from common eye field progenitors, we hypothesized that early NAM treatment increases the efficiency of RO differentiation, particularly in cell lines that initially show poor retinal differentiation capacity.

To test this hypothesis, we treated early retinal differentiation cultures for different durations with NAM and determined RO differentiation efficiency in multiple cell lines. We also examined differentiation marker expression and performed transcriptomic analysis to investigate the mechanism(s) of NAM in this process. We report that NAM treatment for the first 8 days of retinal differentiation significantly increased RO production across multiple cell lines, including the ones that proved to be previously intractable using multiple established protocols. ROs derived with NAM showed a morphology and retinal cell type biogenesis indistinguishable from the controls. Further analyses indicate that NAM treatment favored neural induction over non-neural ectoderm cell fate at the early stage of differentiation and suggest that this effect is mediated, at least in part, through the inhibition of bone morphogenesis protein (BMP) pathway, a well-known anti-neuralizing signal (Am et al., 2012). Overall, our study suggests that early NAM treatment improves RO production yield in general, and importantly, promotes successful differentiation of proven “difficult” lines. Our findings should facilitate the broader use of ROs for disease modeling and therapeutic applications.

## Materials and Methods

### hPSC Generation and Maintenance

The human embryonic stem cell (hESC) line is the *CRX*-GFP H9 line generated and characterized as described in our previous publication (Kaewkhaw et al., 2015). All hiPSC lines (hiPSC1-7) were reprogrammed from fibroblasts isolated from skin biopsies using integration-free Sendai virus carrying the four Yamanaka factors as previously described (Beers et al., 2015) and selected as previously described (Singh et al., 2021). Pluripotency was confirmed by immunostaining of multiple pluripotency markers SSEA4, OCT4, TRA-1-60 and NANOG (data not shown). Donor information was summarized in Supplementary Table 1 and the identity of each cell line was confirmed by STR profiling (Supplementary Figure 1). All hPSC lines were maintained on growth factor-reduced (GFR) or hESC-qualified Matrigel (Corning, NY)-coated plates using either Essential 8 (E8; ThermoFisher Scientific, Waltham, MA) or mTeSR1 medium (Stemcell Technologies, Vancouver, Canada) and were passaged at 60–80% confluency using the EDTA-based method. hPSC lines between passage 20 and 25 were used for RO differentiation, except H9, which was used between passage 80 and 85.

### Retinal Organoid Quantification

The number of ROs in 3 independent differentiation batches was quantified based on morphological criteria (presence of a phase-bright neuroepithelial outer rim). Comparisons between non-treated and NAM-treated conditions were performed using an unpaired Student's *t*-test and statistical significance was defined as *p* < 0.05.

### Image Acquisition Analysis and Quantification

Brightfield images were taken using an EVOS XL Core Cell Imaging System (ThermoFisher Scientific, Waltham, MA). Fluorescence images were acquired with LSM-700 or LSM-880 confocal microscope (Zeiss, Oberkochen, Germany) with Zen software. Fiji and Photoshop CC 2019 software were used for image export, analysis and processing. Comparisons between non-treated and NAM-treated conditions were performed using an unpaired Student's *t*-test and statistical significance was defined as *p* < 0.05.

### RNA Extraction and mRNA Sequencing Library Preparation

Total RNAs were extracted using RNeasy Plus Mini kit (Qiagen, Germantown, MD). At least 3 ROs from different batches were used for each biological replicate. Quality of isolated RNAs was assessed using Bioanalyzer RNA 6000 nano assays (Agilent, Santa Clara, CA) and only high-quality RNAs (RNA integrity number (RIN) > 8) were used for construction of mRNA sequencing library. One hundred nanogram of total RNA were used to construct the strand-specific libraries using TruSeq Stranded mRNA Library Prep Kit-v2 (Illumina, San Diego, CA) with slight modifications as previously described (Brooks et al., 2012). A total of 16 samples were used and they were comprised of two biological replicates from untreated and NAM-treated hiPSC1 and hiPSC3 at two time points (D4, D7).

## Results

### Early NAM Treatment Improves RO Yield

**Figure 1:**
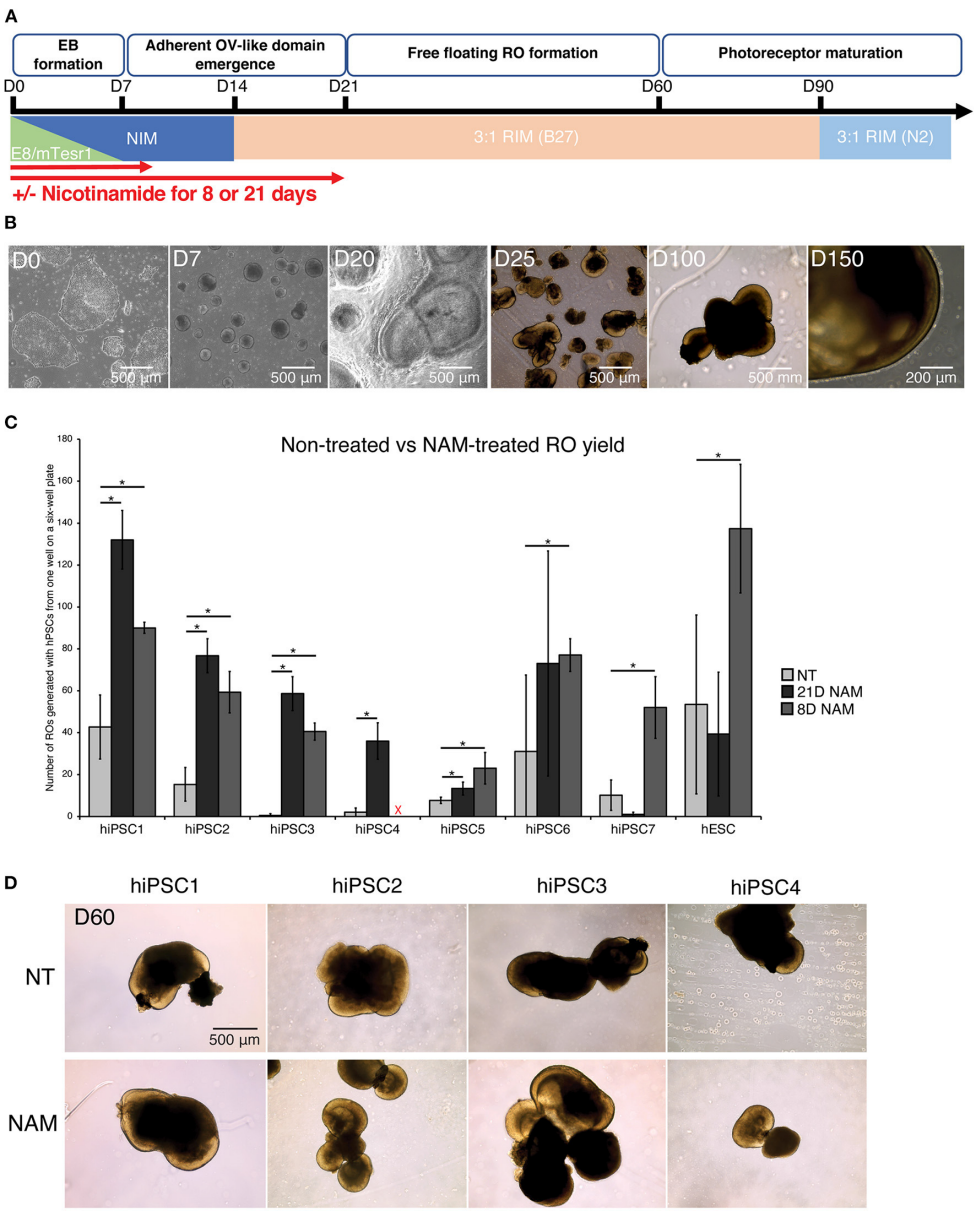
Nicotinamide treatment increased retinal organoid (RO) yield across multiple cell lines. **(A)** Schematic diagram of RO differentiation from human pluripotent stem cells (hPSCs). EBs, Embryoid Bodies; ROs, Retinal Organoids; NIM, Neural Induction Medium; RIM, Retinal Induction Medium; OVs, Optic Vesicles. **(B)** Typical morphology of ROs at different time points of the differentiation. **(C)** Comparison of RO production at D60 between non-treated (NT) cultures and cultures treated with nicotinamide (NAM) for 21 or 8 days. NAM, Nicotinamide. The red cross indicates lack of data for hiPSC4 under 8D NAM treatment. **(D)** Brightfield images of ROs at day 60 produced without NAM (upper panel), or with 21-day (middle panel) or 8-day (lower panel) NAM treatment. The number of ROs in 3 independent differentiation batches were quantified. *p < 0.05; n.s., non-significant.

To assess the effect of NAM treatment on RO yield, we differentiated 1 hESC line and 7 hiPSC lines with and without 5 mM NAM supplementation for different durations (8 vs. 21 days) (Figure 1A). All hPSC lines were able to differentiate into retinal organoids with typical morphology (Figure 1B).

The number of ROs generated with and without NAM treatment was quantified at D60 based on morphological criteria (presence of a phase-bright neuroepithelial outer rim). Substantial variation in differentiation capacity was observed across hiPSC lines. While some lines displayed high efficiency in RO production without NAM treatment (e.g., >40 ROs/batch in hiPSC1), others such as hiPSC3 produced <1 RO/batch on average (Figure 1C). NAM treatment between D1 and D20 significantly increased RO production in most of the hiPSC lines (hiPSC1, 2, 3, 4, and 6; 3.0–117.3-fold increase). In particular, 3 cell lines with limited differentiation capacity (generating <10 organoids per batch of differentiation) in the untreated condition responded significantly to NAM treatment with, for example, a 117.3-fold increase in RO production for hiPSC3. However, we also noted some deleterious effects of NAM treatment in one cell line (hiPSC7; 10.2-fold decrease) (Figure 1C). We hypothesized that, given the complex and varied effects of NAM, prolonged treatment could offset some of the early benefits and indeed could be deleterious. We therefore investigated if a shorter NAM treatment from D1 to D8 could avoid the adverse effects on those cell lines while maintaining the improvement of RO production on other cell lines. A shorter treatment significantly and consistently improved the yield of RO in the tested cell lines, including the ones that previously showed little or even negative response to the 21-day treatment regime (Figure 1C, e.g., hiPSC7 and hESC, 5.1-fold and 2.6-fold increase, respectively, in D8 treatment). We concluded that NAM treatment for the first 8 days of differentiation was sufficient to significantly increase the yield of RO while prolonged treatment may negatively impact later stages of retinal differentiation efficiency in some cell lines. No morphological difference was observed between untreated and NAM-treated ROs and even a prolonged NAM treatment did not alter size or morphology of the organoids generated compared to controls (Figure 1D).

### NAM-ROs Show Similar Morphology and Biogenesis of Major Retinal Cell Types to the Control

**Figure 2:**
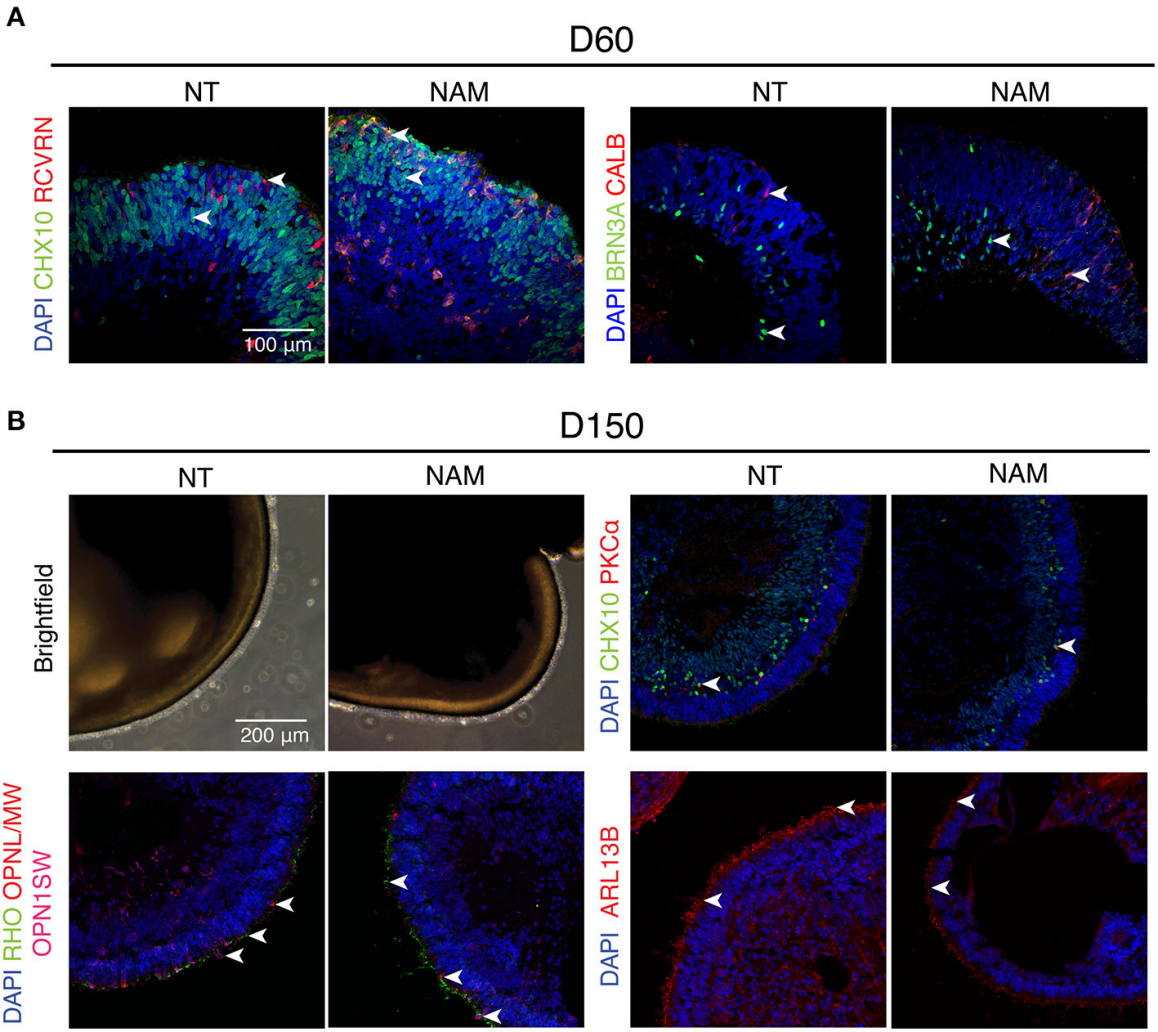
Non-treated (NT) and Nicotinamide (NAM)-treated retinal organoids displayed similar biogenesis of major retinal cell types at differentiation day 60 and 150. **(A)** Immunohistochemistry analysis of human induced pluripotent stem cell (hiPSC)1-derived ROs at D60 using antibodies against markers for retinal progenitor or bipolar cells (CHX10, green), photoreceptor progenitor cells (RCVRN, red), retinal ganglion cells (BRN3A, green) and horizonal or amacrine cells (CALB, red). **(B)** Immunohistochemistry analysis of hiPSC1-derived ROs at D150 using antibodies against markers for rod bipolar cells (PKCα, red), retinal progenitor or bipolar cells (CHX10, green), cone photoreceptors (OPN1SW, magenta; OPNL/MW, red), rod photoreceptors (RHO, green) and cilia (ARL13B, red). Nuclei were stained with 4′,6-diamidino-2-phenylindole (DAPI, blue). Arrowheads indicate relevant staining of each marker.

To determine whether ROs obtained by NAM treatment differentiated similarly to non-treated ones, we assessed the biogenesis of major retinal cell types at different time points (Figure 2). As expected, a thick layer of retinal progenitor cells (CHX10+) was observed in both control and treated ROs at D60, when Recoverin (RCVRN+) photoreceptor progenitor cells start to emerge. Retinal ganglion cells (RGCs; BRN3A+) and horizontal cells (CALB+) were also present in abundance in organoids by that time regardless of NAM treatment (Figure 2A).

Robust expression of rhodopsin (RHO) and cone opsins (L/M and S-opsin) highlighted the emergence of rod and cone photoreceptors under both conditions at D150 (Figure 2B). Moreover, formation of outer segment-like structure, as shown by the presence of brush-like structures protruding out of the ROs in the phase-contrast images, and the expression of ciliary marker ARL13B (Figure 2B and Supplementary Figure 2), revealed no significant difference in photoreceptor maturation in both untreated and treated cultures. Besides, birth of bipolar cells in both organoids was evidenced by the expression of CHX10 (pan-bipolar cell marker) and PKCα (rod bipolar cell marker) (Figure 2B and Supplementary Figure 2). Taken together, these results show that early NAM treatment does not affect subsequent differentiation of major retinal cell types, including the maturation of photoreceptor cells.

### NAM Treatment Promotes Early Differentiation of Eye Field Progenitors

**Figure 3:**
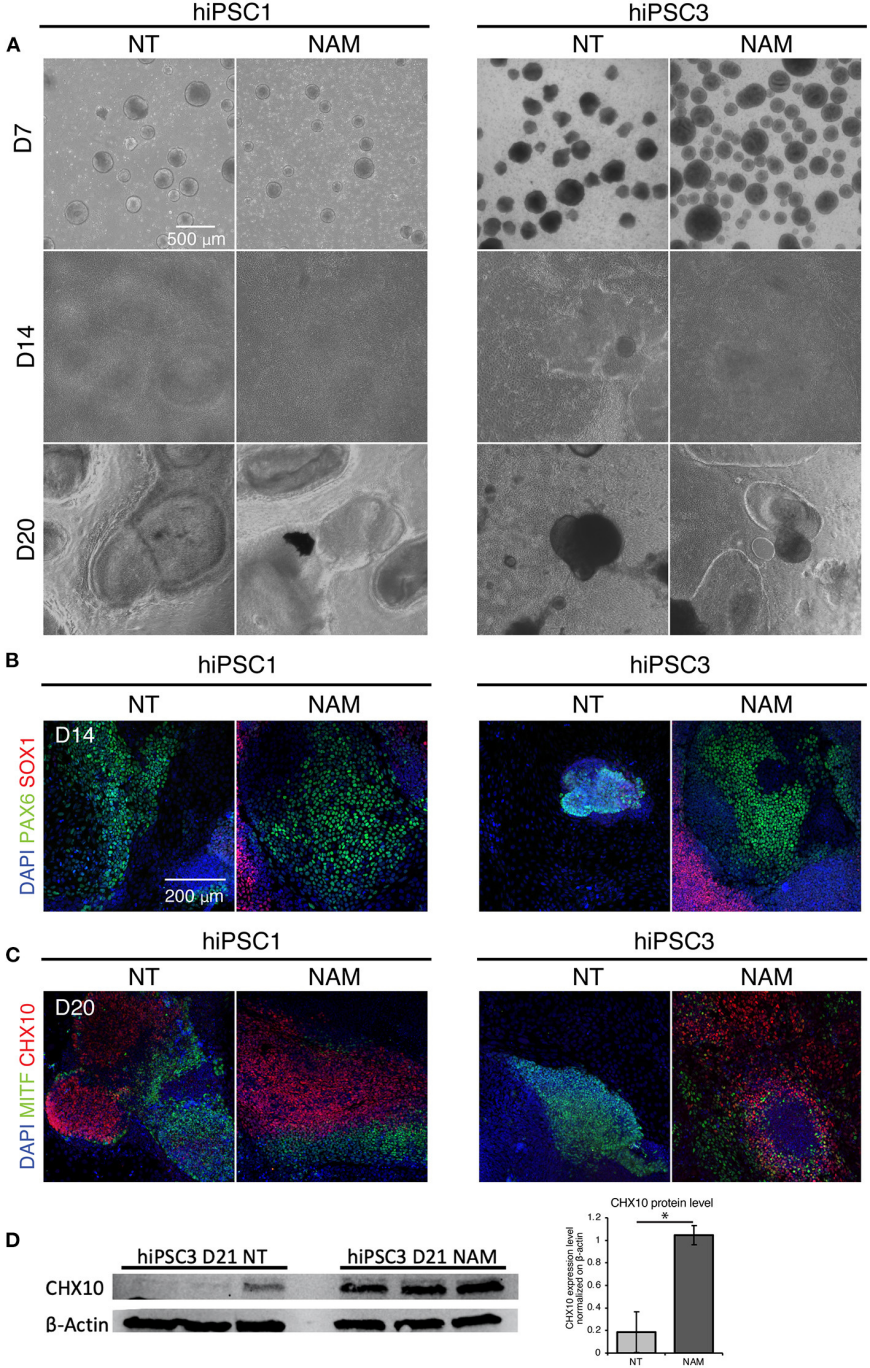
Nicotinamide treatment improves EB formation and eye field progenitor differentiation and spreading. **(A)** Bright field images of non-treated (NT) and Nicotinamide-treated cultures at differentiation day 7, 14, 20. **(B)** Immunostaining of neuroectodermal makers (PAX6/SOX*1*). **(C)** Retinal markers CHX10 and MITF showing neural retinal progenitor cells and prospective pigmented epithelial cells, respectively. **(D)** Western blot analysis (left) and quantification (right) of the expression of the neural retina progenitor marker CHX10 in NT and NAM-treated hiPSC3 cultures at D21. 3 independent differentiation batches were quantified. *p < 0.05.

We then investigated NAM mechanism of action in hiPSC1 and hiPSC3 lines, which were selected as typical examples of a high and low yield line, respectively. We first compared the morphology of non-treated hiPSC1 and hiPSC3 (Figure 3A, left panel). Morphological difference in EBs could be observed as early as D7, suggesting differences in neural cell fate commitment. hiPSC1 line generated spherical and compact EBs while hiPSC3 produced cell aggregates with polymorphic appearances (Figure 3A). One day after plating, both hiPSC1 and hiPSC3 EBs attached to Matrigel-coated dishes. However, in contrast to hiPSC1 EBs that quickly flattened and expanded, EBs generated from hiPSC3 failed to expand and cover the entire culture dish even by D14. OV-like structures started to emerge in adherent culture of hiPSC1 at D20, while mostly fibroblast-like cells were present in hiPSC3-derived cultures (Figure 3A). We then evaluated the effect of NAM treatment on cell morphology. While no adverse effects were observed on hiPSC1 cells, EBs generated from NAM-treated hiPSC3 clearly appeared more spherical and compact than non-treated ones, suggesting NAM was able to improve neural cell fate commitment of these cells. Subsequently, these EBs were able to adhere and spread out comparably to those generated from hiPSC1 line and formed OV-like structures at D20, while the proportion of fibroblast-like cell area was reduced compared to the non-treated condition (Figure 3A).

To confirm that NAM treatment favors neural and eye field commitment, we evaluated the expression of anterior neuroectoderm (SOX1), eye field (PAX6), NR (CHX10) and RPE (MITF) markers identified in previous study (Zhong et al., 2014) at D14 and D20 by immunostaining. Retinal differentiation induced the formation of large prospective eye field domains (PAX6+/SOX1–) at D14 in non-treated hiPSC1-derived cultures (Figure 3B), which likely later form the presumptive NR (CHX10+) and RPE (MITF+) domains observed at D20 (Figure 3C). In contrast, few potential eye-field domains were found in non-treated hiPSC3-derived cultures at D14 and the expression of PAX6 was restricted to small area corresponding to EBs that failed to spread out (Figure 3B). Consequently, no presumptive NR domains and very few small RPE domains were observed in hPSC3-derived cultures at D20 (Figure 3C and Supplementary Figure 3). While no major difference was found in the expression of neural and eye field markers in hiPSC1 cultures following NAM treatment, NAM promoted the formation of large eye field domains in hiPSC3-derived culture at D14, which subsequently differentiated into presumptive RPE and NR domains at D20 (Figures 3B,C, and Supplementary Figure 3). An increased expression of the NR progenitor marker CHX10 in NAM treated hiPSC3 culture at D21 was confirmed by Western blot analysis (Figure 3D). Taken together, these data suggest that NAM treatment can improve RO yield by promoting early neural/eye field commitment.

### NAM Improves Retinal Differentiation at Least in Part Through BMP Pathway Inhibition

**Figure 4:**
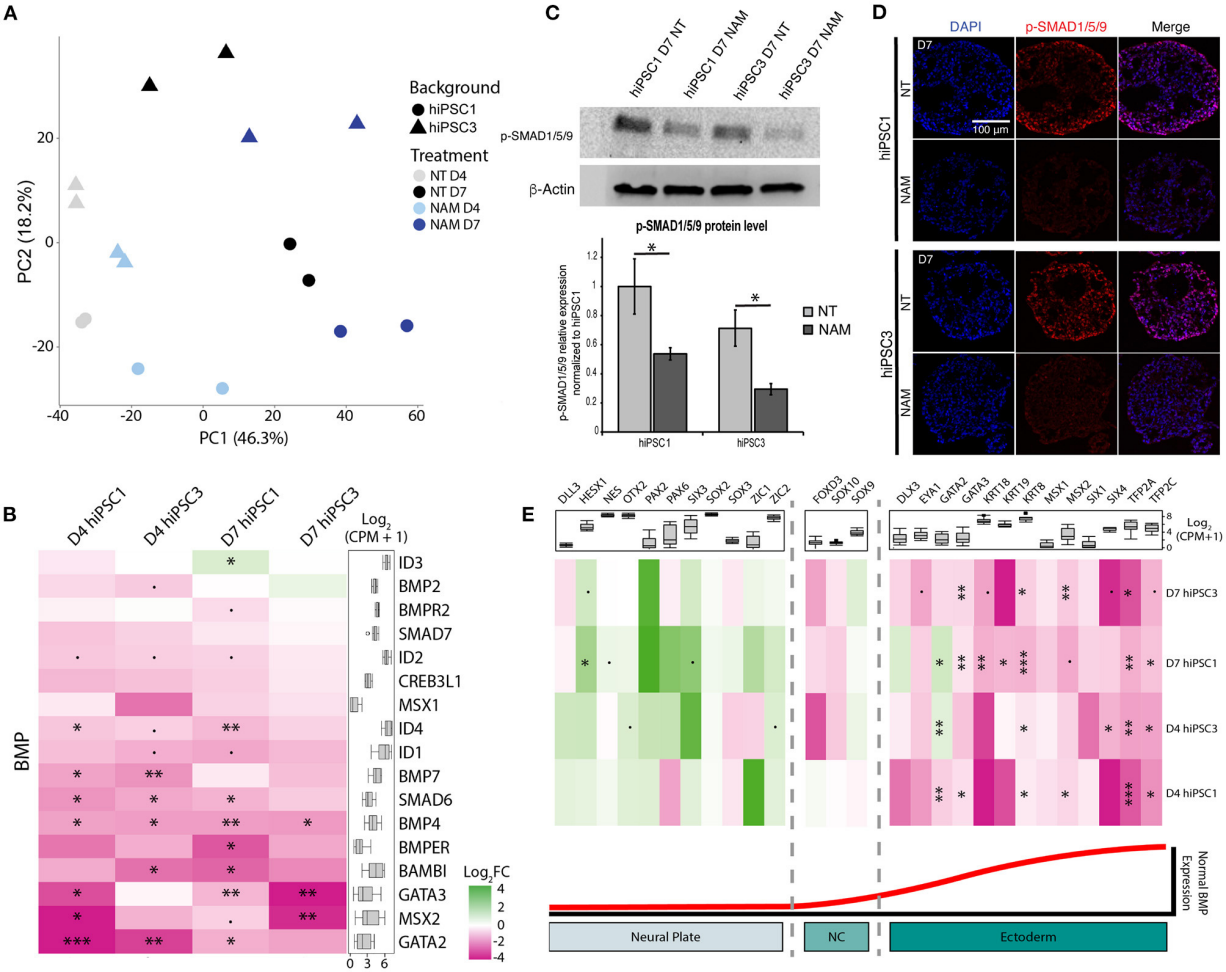
Transcriptomic analysis reveals that nicotinamide treatment inhibits BMP pathway and improves neuroectodermal differentiation. **(A)** Principal component analysis (PCA) plot of non-treated (NT) and Nicotinamide-treated (NAM) hiPSC1 and hiPSC3-derived EBs samples based on log2 CPM gene counts. **(B)** Log2 fold change (FC) heatmap comparing the expression of BMP ligands and BMP target genes between non-treated and NAM-treated EBs at D4 and D7 (shown as NAM-treated vs. non-treated). **(C)** Western blot analysis (top) and quantification (bottom) of the phosphorylated form of SMAD1/5/9 (Key BMP pathway effectors) in NT and NAM-treated cultures at D7. 3 independent differentiation batches were quantified. *p < 0.05. **(D)** Immunohistochemistry analysis of the expression of the phosphorylated form of SMAD1/5/9 in NT and NAM-treated cultures at D7. **(E)** Log2 fold change (FC) heatmap comparing the expression of neural and non-neural ectoderm markers between non-treated and NAM-treated EBs at D4 and D7 (shown as NAM-treated vs. non-treated). The box plot shows the minimum, first quartile, median, third quartile, and maximum of count per million (CPM) in each category. •*p* < 0.1; *p < 0.05; **p < 0.01; ***p < 0.05.

To investigate how NAM modulates cell fate commitment, we characterized the gene expression profiles of D4 and D7 EBs generated from hiPSC1 and hiPSC3 lines with and without NAM. Despite variation between cell lines, principal component analysis revealed a similar temporal progression and a similar response to NAM treatment in EBs derived from both cell lines, as shown by PC1 and PC2, respectively (Figure 4A). Interestingly, analysis of the differentially expressed (DE) genes revealed decreased expression of key components of the BMP pathway including *BMP4, BMP7*, and *SMAD6* in both cell lines at D4 and D7 following NAM treatment. To further explore the effect of NAM on the activity of this pathway, a gene set consisting of the secreted BMP molecules BMP2/4/7 and well-known BMP target genes was created based on published literature (Karaulanov et al., 2004; Miyazono et al., 2005). Log2 fold change heat map analysis generated from this gene set revealed the downregulation of most of the BMP target genes (e.g., *GATA3, BAMBI* and *ID*s) in NAM-treated EBs (Figure 4B). Gene set enrichment analysis (GSEA) also validated the overall downregulation of the expression of these genes in both hiPSC1 and hiPSC3 lines, strongly suggesting that NAM treatment inhibits BMP signaling during early retinal differentiation of hPSCs. To further validate the reduction of BMP activity in NAM-treated cultures, we confirmed the decreased protein levels of phosphorylated SMAD1/5/9, a key effector of the BMP pathway, at D7, by Western blot analyses and immunostaining (Figures 4C,D, respectively).

In vertebrates, BMP signaling mediates epidermal induction and its inhibition in the ectoderm is a hallmark of neural-fate acquisition. To evaluate if NAM treatment favors neural commitment of hPSCs, two other sets of genes consisting of early neural and non-neural ectoderm markers described in the literature were created (Groves and LaBonne, 2014; Qu et al., 2016). GSEA indicated a significant enrichment of transcripts associated with neural specification and a reduced expression of non-neural ectoderm-associated genes in both cell lines at D7 following NAM treatment (Figure 4E). Log2 fold change heat map analysis confirmed that NAM treatment had a tendency to increase the expression of early neural markers, such as *OTX2, PAX2*, and *SIX3*, while significantly decreasing the expression of non-neural ectoderm ones including *TFAP2A/C, GATA3*, and *KRT8/18/19*. Taken together, these results suggest that NAM treatment promotes neural commitment of hPSCs at the expense of non-neural ectoderm cell fate through the inhibition of BMP signaling. This effect of NAM underlies, at least in part, the increased yield of RO generation.

## Discussion

One of the most significant hurdles to the broader use of RO technology for disease modeling is the variability of differentiation capacity across different cell lines, with some lines consistently performing poorly under current protocols (Chichagova et al., 2020; Cowan et al., 2020). In this study, we identified NAM as an extrinsic factor to improve RO production when applied at an early stage of differentiation. The beneficial effect is particularly pronounced in the lines with low initial efficiency, making them consistently well performing.

Genetic and epigenetic heterogeneities among hPSC lines impact their capacity to differentiate into desired cell types *in vitro*. Under well-controlled experimental pipelines, it was found that donor variations are the largest contributor to the heterogeneity of hiPSCs (Kilpinen et al., 2017). Yet, we note that various experimental factors also greatly impact the genetic and/or epigenetic properties of hiPSCs. Although different reprogramming methods reset the epigenetic markers to various extent, epigenetic memory is commonly found in hiPSCs generated using Yamanaka factors (Kim et al., 2010). Consequently, iPSCs reprogrammed from mouse retinal neurons have been demonstrated to have a higher differentiation efficiency into retinal organoids compared to those generated from fibroblasts due to the retention of retinal-specific epigenetic markers (Hiler et al., 2015). As such, the number of passages might also impact the efficiency of differentiation, as a low passage hiPSCs could retain more epigenetic markers associated with the original cell type used for reprogramming or retain partial expression of the Yamanaka factors compared to those in higher passages. On the other hand, hiPSCs tend to accumulate chromosomal abnormalities during passaging and thus excessively high passages could negatively impact the differentiation efficiency and later applications (Kim et al., 2010; Hayashi et al., 2019). In our study, we strictly control the reprogramming method and passages for hiPSCs and employ an identical passage protocol for an objective comparison of multiple cell lines. We identified a failure to commit to early neural fate as one of the reasons for poor differentiation capacity in hiPSC3 line. This could be due to individual genetic variations among study subjects, or genetic and/or epigenetic modifications acquired in cultures despite our standardized pipeline. Our study suggests that the variation in RO differentiation efficiency between cell lines can be overcome by supplementation of NAM into early differentiation cultures and that NAM favored neural cell fate commitment, at least in part, through the inhibition of the BMP signaling pathway. *In vivo*, BMP ligands signal ectoderm cells to differentiate into non-neural epithelial cells, while the action of secreted BMP inhibitors, such as Noggin and Chordin, is required for neural plate formation (Muñoz-Sanjuán and Brivanlou, 2002; Qiao et al., 2012). Consistently, variations in endogenous BMP signaling level was previously shown to impact the ability of hiPSCs to differentiate into neural crest and non-neural ectoderm-derived cells (Hackland et al., 2017). Such intrinsic variation in BMP signaling activities may also partially explain the differences in neural commitment efficiency across hiPSC lines.

Previous studies found that a short BMP4 treatment from D6 onwards promoted RO production and decreased telencephalic marker *FOXG1* expression (Kuwahara et al., 2015; Capowski et al., 2019), suggesting that BMP signaling at a later stage of RO differentiation is required to prevent hPSC-derived neuroepithelium from acquiring a telencephalic fate. Consistently, we found that prolonged NAM treatment was deleterious for retinal differentiation in some hiPSC lines. As progression of retinal differentiation may vary depending on specific protocols, we note that the optimal chronological age for NAM withdrawal could also vary by a few days. Consequently, a shorter 4/5-day treatment could be optimal in some cases.

Importantly, NAM is known to impact numerous other cellular processes and signaling pathways that probably also participate in its effect on neural and eye field commitment. NAM is the amide form of vitamin B3 and a precursor of nicotinamide adenine dinucleotide (NAD), an essential cofactor involved in ATP production and DNA repair (Surjana et al., 2010). It has been shown that NAM rescues hESC-derived neuroectoderm from the abundant cell death generally observed at the beginning of neural differentiation though inhibition of Poly [ADP-ribose] polymerase 1 (Cimadamore et al., 2009). Thus, NAM treatment could possibly also improve the yield of ROs by reducing cell death during the initial steps of differentiation. Additionally, previous studies also reported that NAM favors neural differentiation through inhibition of sirtuins and increases the expression of EF makers in hESC derived-adherent cultures through CK1 inhibition (Meng et al., 2018). Further investigations are required to fully understand how NAM modulates early RO differentiation.

To conclude, our study demonstrates that NAM promotes early hPSC neural fate commitment and improves RO production. Supplementation of NAM in current retinal differentiation protocols increases the yield of ROs in general but has a particularly beneficial impact on consistently “difficult” hPSC lines. Our findings will facilitate a broader use of this *in vitro* model to study retinal degenerative diseases and to develop therapies.

## Data Availability Statement

The datasets presented in this study can be found in online repositories. The name of the repository and accession number can be found at: National Center for Biotechnology Information (NCBI) Gene Expression Omnibus (GEO), https://www.ncbi.nlm.nih.gov/geo/, GSE164884.

## Author Contributions

FR and TL: conceptualization. FR, ZB, and TL: methodology. FR and ZB: data analysis. FR, HC, LG, and RK: investigation. FR and HC: writing—original draft. FR, HC, ZB, AS, and TL: writing—review and editing. TL and AS: funding acquisition and resources. TL: supervision. All authors contributed to the article and approved the submitted version.

## Funding

This research was supported by Intramural Research Programs of the National Eye Institute (ZIAEY000490, ZIAEY000474, and ZIA000546), and Mr. Yair Mendels through the USHER2020 Foundation.

## Conflict of Interest

The authors declare that the research was conducted in the absence of any commercial or financial relationships that could be construed as a potential conflict of interest.

## Publisher's Note

All claims expressed in this article are solely those of the authors and do not necessarily represent those of their affiliated organizations, or those of the publisher, the editors and the reviewers. Any product that may be evaluated in this article, or claim that may be made by its manufacturer, is not guaranteed or endorsed by the publisher.
