# Supplementary material for: Nicotinamide Promotes Formation of Retinal Organoids From Human Pluripotent Stem Cells via Enhanced Neural Cell Fate Commitment
Source: Front Cell Neurosci. 2022 Jun 17;16:878351. doi: 10.3389/fncel.2022.878351 (PMC9247291; doi:10.3389/fncel.2022.878351)
Supplement: Supplementary file 1 [file Data_Sheet_1.docx]

**Supplemental Experimental Procedures**

**Retinal organoid (RO) differentiation**

RO differentiation was performed as previously described (Regent et al., 2020) (Figure 1A). Briefly, after dissociated into small clumps using 0.5 mM EDTA/PBS (Corning, NY), hPSCs from one well of a 6-well plate were resuspended in either E8 or mTeSR1 medium supplemented with 10 µM Y-27632 (Tocris, Bristol, United Kingdom) and transferred into one 100‐mm polyHEMA (Sigma, St. Louis, MO)-coated petri dish for embryoid body (EB) formation. At Day (D) 1 and D2, neural induction medium (NIM: DMEM/F-12 (1:1) (ThermoFisher Scientific, Waltham, MA) supplemented with 1% N2 supplement (ThermoFisher Scientific, Waltham, MA), 1x MEM non-essential amino acids (NEAA; Sigma, St. Louis, MO), and 2 µg/ml heparin (Sigma, St. Louis, MO)) was added at a ratio of 3:1 and 1:1, respectively. Media were switched to full NIM media at D3. D7 EBs from one 100-mm petri dish were plated onto a 60-mm tissue culture-treated dish coated with GFR Matrigel (Corning, NY) and cultured in neural induction medium (NIM), with media changed every 2-3 days. At D14, NIM were switched to 3:1 retinal induction medium (3:1 RIM: DMEM/F-12 (3:1) supplemented with 2% B27 supplement without Vitamin A (ThermoFisher Scientific, Waltham, MA), 1% antibiotic-antimycotic solution (ThermoFisher Scientific, Waltham, MA), 1% GlutaMAX (ThermoFisher Scientific, Waltham, MA) and 1X NEAA (Sigma, St. Louis, MO)) and media were changed every 2-3 days until D21.

For NAM treatment, a final concentration of 5mM NAM (Sigma, St. Louis, MO) was added to NIM and 3:1 RIM culture media from D1 to D8 or D21 (Figure 1A). NAM (Sigma, St. Louis, MO) was dissolved in ultrapure water to a final concentration of 1M. Ultrapure water was used as control.

At D21, adherent cells from one 60-mm dish were scraped off into small clumps (<5 mm^2^) and transferred to two polyHEMA-coated 100-mm petri dishes. Resulting floating clumps were cultured in 3:1 RIM supplemented with 20 ng/ml insulin-like growth factor 1 (IGF-1; ThermoFisher Scientific, Waltham, MA) and 1 mM taurine (Sigma, St. Louis, MO). Full-media change was performed the next day to remove cellular debris. Around D28, well-formed OVs characterized by a phase-bright outer rim were isolated from non-retinal tissues using 30-gauge needles and transferred to new polyHEMA-coated culture dishes using a wide-bore pipette.

From D35 onward, 10% fetal bovine serum (FBS; ThermoFisher Scientific, Waltham, MA) was added to 3:1 RIM supplemented with 20 ng/ml IGF-1, and 1 mM taurine. Starting from D63, 3:1 RIM was supplemented with 1 mM 9-*cis* retinal. 1% N2 supplement (ThermoFisher Scientific, Waltham, MA) was used instead of B27 supplement without Vitamin A and concentration of 9-*cis* retinal was reduced to 0.5 mM from D91 till the end of differentiation. Media were half changed every 2-3 days, with IGF1, taurine and 9-*cis* retinal freshly added to the media under dim light environment.

**Immunofluorescence**

EBs and adherent cells cultured on chamber slides (ThermoFisher Scientific, Waltham, MA) were fixed using 4% paraformaldehyde (FD NeuroTechnologies, Columbia, MD) for 10 min at room temperature, followed by 3 washes with PBS. ROs were fixed in 2% paraformaldehyde for 1 hour at room temperature. EBs and ROs were then cryoprotected using a sucrose gradient (15-30%) and embedded in M1 embedding matrix (Thermo Scientific, Waltham, MA). A Leica Biosystems Cryostat (Leica Biosystems, Buffalo Grove, IL) was used to obtain 10µm sections, which were dried on Superfrost Plus Microscope slides (Fisher Scientific, Hampton, NH) before storage at -20°C until use. For immunostaining, sections and adherent cells were incubated in blocking solution (PBS with 10% donkey serum and 0.1% Triton) for 30 mins at room temperature prior to overnight incubation with primary antibodies at 4 °C (Antibody information and dilution are listed in Supplementary Table 2). After three 10-min washes in PBS, appropriate Alexa Fluor-conjugated secondary antibodies and 4′,6-diamidino-2-phenylindole (DAPI; Invitrogen, Carlsbad, CA) were added for 1 hour at room temperature.

**Western blot analysis**

EBs were plated on Matrigel-coated 12-well plates and harvested at D21 in 80 μl of RIPA buffer (Sigma) supplemented with 1x protease inhibitors (Roche) and 1x phosphatase inhibitor (Roche) using cell scrapers. The lysate was incubated on ice for half an hour and centrifuged at 10,000 *g* for 10 minutes at 4 °C. The supernatant was either stored at -80 °C until use or quantified by Pierce bicinchoninic acid (BCA) protein assay (ThermoFisher Scientific). Approximately 20 μg protein was diluted 4:1 in reducing 4X Laemmeli buffer (Biorad) and boiled for 10 minutes. The samples were separated at 150-200 V for 1 hour on 4-15% precast polyacrylamide gel (Biorad) and transferred to polyvinylidene fluoride (PVDF) membranes using TransBlot® Turbo™ Transfer System (Biorad). After blocking in 5% bovine serum albumin (BSA) for 1 hour at room temperature, the blots were incubated in antibodies with appropriate dilutions (Supplemental Table 2) overnight in 1% BSA in 1X TBS-T at 4 °C overnight with gentle agitation. Membranes were subsequently washed in 1X TBS-T for 3 times, 10 minutes each, and incubated in 1X TBS-T with horseradish peroxidase-conjugated secondary antibodies (1:5000) for 1 hour at room temperature, followed by another three 10-minutes wash. Before imaging, the membranes were exposed to SuperSignal® West Pico enhanced chemiluminescence (ECL) solution (ThermoFisher Scientific) for 5 minutes, and chemiluminescence was captured using a Bio-Rad ChemiDoc™ touch (Bio-Rad).

**RNA-seq analysis**

Libraries were sequenced on two lanes of an Illumina HiSeq 2500 (San Diego, CA) in paired-end mode (125 bp read length). Reads passing the Illumina chastity filter were demultiplexed then trimmed to remove low quality bases and adapters using Trimmomatic v0.36 (Bolger et al., 2014). Next, reads were aligned to the human transcriptome (GRCH38.p13, Ensembl 98) and quantified to the transcript-level using Kallisto v0.45.0 (Bray et al., 2016).

All further analyses were completed in R Studio (RStudio Team, 2015). Transcript counts were summarized to the gene level using tximport 1.14.2 with the lengthScaledTPM option (Soneson et al., 2015) and TMM normalized using edgeR v3.28.1 (McCarthy et al., 2012; Robinson et al., 2010). Genes with at least 1 count per million reads (CPM) in both replicates of at least one group (e.g., both D4 hiPSC3 samples) were retained for downstream analysis. Principal component analysis (PCA) was performed using the prcomp function from the stats v3.6.2 package (R Core Team, 2019) on log2 CPM gene counts. The edgeR-limma workflow (Law et al., 2016) used to assess differential expression between NAM treated and untreated samples by time and cell line; genes with a log2 fold change >1 and false discovery rate (FDR) of <0.05 were identified as differentially expressed.

Gene set enrichment analysis (GSEA) was performed with fgsea v1.12.0 (Korotkevich et al., 2019) using the GO gene sets from MSigDB v7.1 (Liberzon et al., 2011; Subramanian et al., 2005) supplemented with custom gene sets of interest selected based on previous publications. PCA, heatmaps, and enrichment plots were generated using ggplot2 v3.3.0, ComplexHeatmap v2.2.0 and fgsea v1.12.0, respectively (Gu et al., 2016; Korotkevich et al., 2019; Wickham, 2009).

**References**

Beers, J., Linask, K.L., Chen, J.A., Siniscalchi, L.I., Lin, Y., Zheng, W., Rao, M., and Chen, G. (2015). A cost-effective and efficient reprogramming platform for large-scale production of integration-free human induced pluripotent stem cells in chemically defined culture. Sci Rep *5*, 11319.

Bolger, A.M., Lohse, M., and Usadel, B. (2014). Trimmomatic: a flexible trimmer for Illumina sequence data. Bioinformatics *30*, 2114–2120.

Bray, N.L., Pimentel, H., Melsted, P., and Pachter, L. (2016). Near-optimal probabilistic RNA-seq quantification. Nat. Biotechnol. *34*, 525–527.

Gu, Y., Li, T., Ding, Y., Sun, L., Tu, T., Zhu, W., Hu, J., and Sun, X. (2016). Changes in mesenchymal stem cells following long-term culture in vitro. Mol Med Rep *13*, 5207–5215.

Kaewkhaw, R., Kaya, K.D., Brooks, M., Homma, K., Zou, J., Chaitankar, V., Rao, M., and Swaroop, A. (2015). Transcriptome Dynamics of Developing Photoreceptors in Three-Dimensional Retina Cultures Recapitulates Temporal Sequence of Human Cone and Rod Differentiation Revealing Cell Surface Markers and Gene Networks. Stem Cells *33*, 3504–3518.

Korotkevich, G., Sukhov, V., and Sergushichev, A. (2019). Fast gene set enrichment analysis. BioRxiv 060012.

Law, C.W., Alhamdoosh, M., Su, S., Dong, X., Tian, L., Smyth, G.K., and Ritchie, M.E. (2016). RNA-seq analysis is easy as 1-2-3 with limma, Glimma and edgeR. F1000Res *5*.

Liberzon, A., Subramanian, A., Pinchback, R., Thorvaldsdóttir, H., Tamayo, P., and Mesirov, J.P. (2011). Molecular signatures database (MSigDB) 3.0. Bioinformatics *27*, 1739–1740.

McCarthy, D.J., Chen, Y., and Smyth, G.K. (2012). Differential expression analysis of multifactor RNA-Seq experiments with respect to biological variation. Nucleic Acids Res. *40*, 4288–4297.

Regent, F., Chen, H.Y., Kelley, R.A., Qu, Z., Swaroop, A., and Li, T. (2020). A simple and efficient method for generating human retinal organoids. Mol. Vis. *26*, 97–105.

Robinson, M.D., McCarthy, D.J., and Smyth, G.K. (2010). edgeR: a Bioconductor package for differential expression analysis of digital gene expression data. Bioinformatics *26*, 139–140.

Soneson, C., Love, M.I., and Robinson, M.D. (2015). Differential analyses for RNA-seq: transcript-level estimates improve gene-level inferences. F1000Res *4*, 1521.

Subramanian, A., Tamayo, P., Mootha, V.K., Mukherjee, S., Ebert, B.L., Gillette, M.A., Paulovich, A., Pomeroy, S.L., Golub, T.R., Lander, E.S., et al. (2005). Gene set enrichment analysis: a knowledge-based approach for interpreting genome-wide expression profiles. Proc Natl Acad Sci U S A *102*, 15545–15550.

Wickham, H. (2009). Toolbox. In Ggplot2: Elegant Graphics for Data Analysis, H. Wickham, ed. (New York, NY: Springer), pp. 65–90.

hiPSC1 hiPSC2 hiPSC3

**
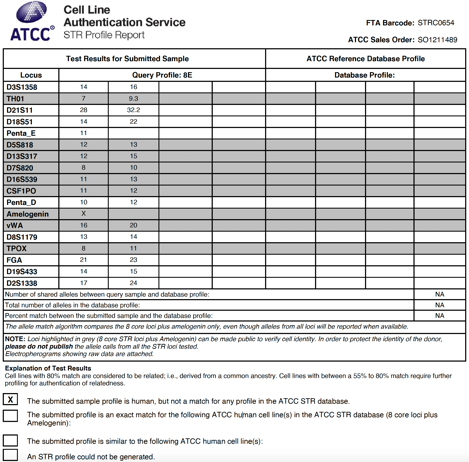

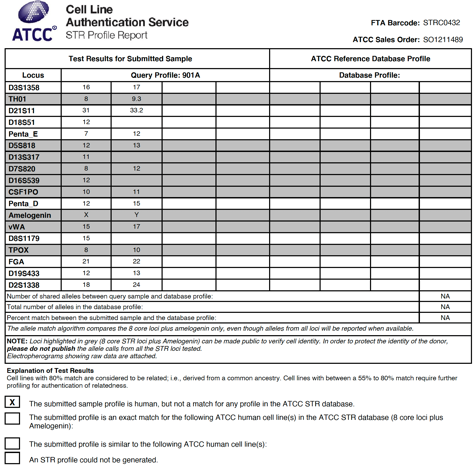
**
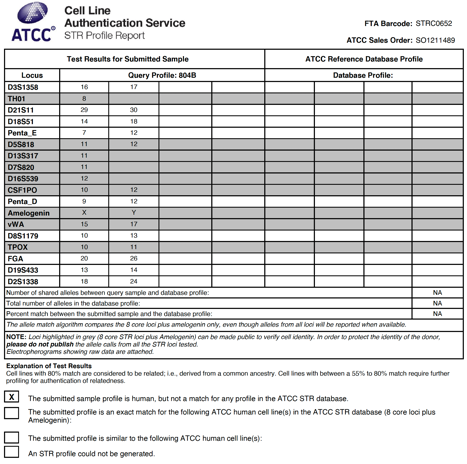


hiPSC4 hiPSC5 hiPSC6

**
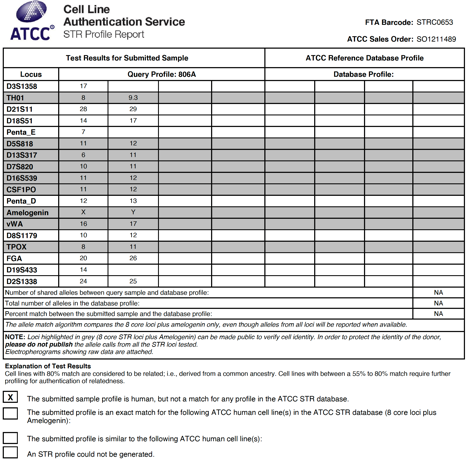
**
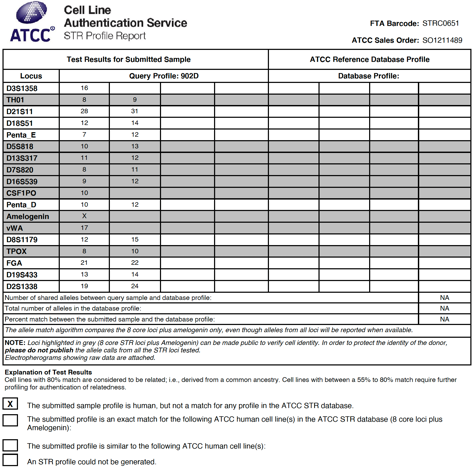

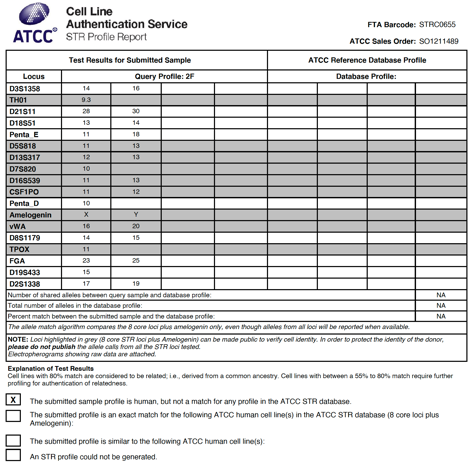


hiPSC7 hESC

**
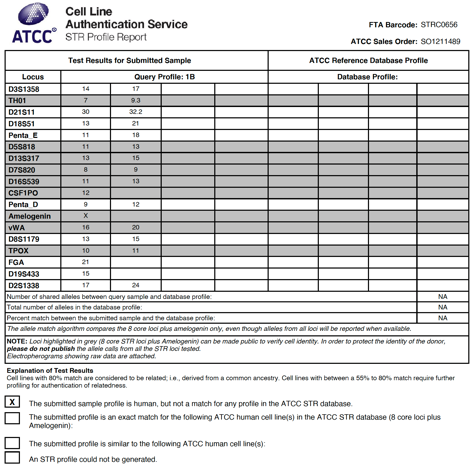

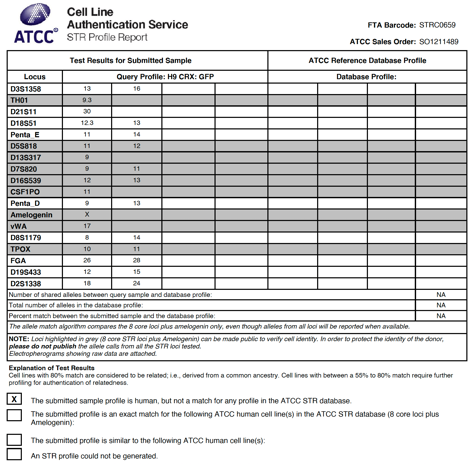
**

**Supplementary Figure 1.** STR profile reports. All 8 human pluripotent stem cell lines used in this study are from different human donors without cross-contamination.

**
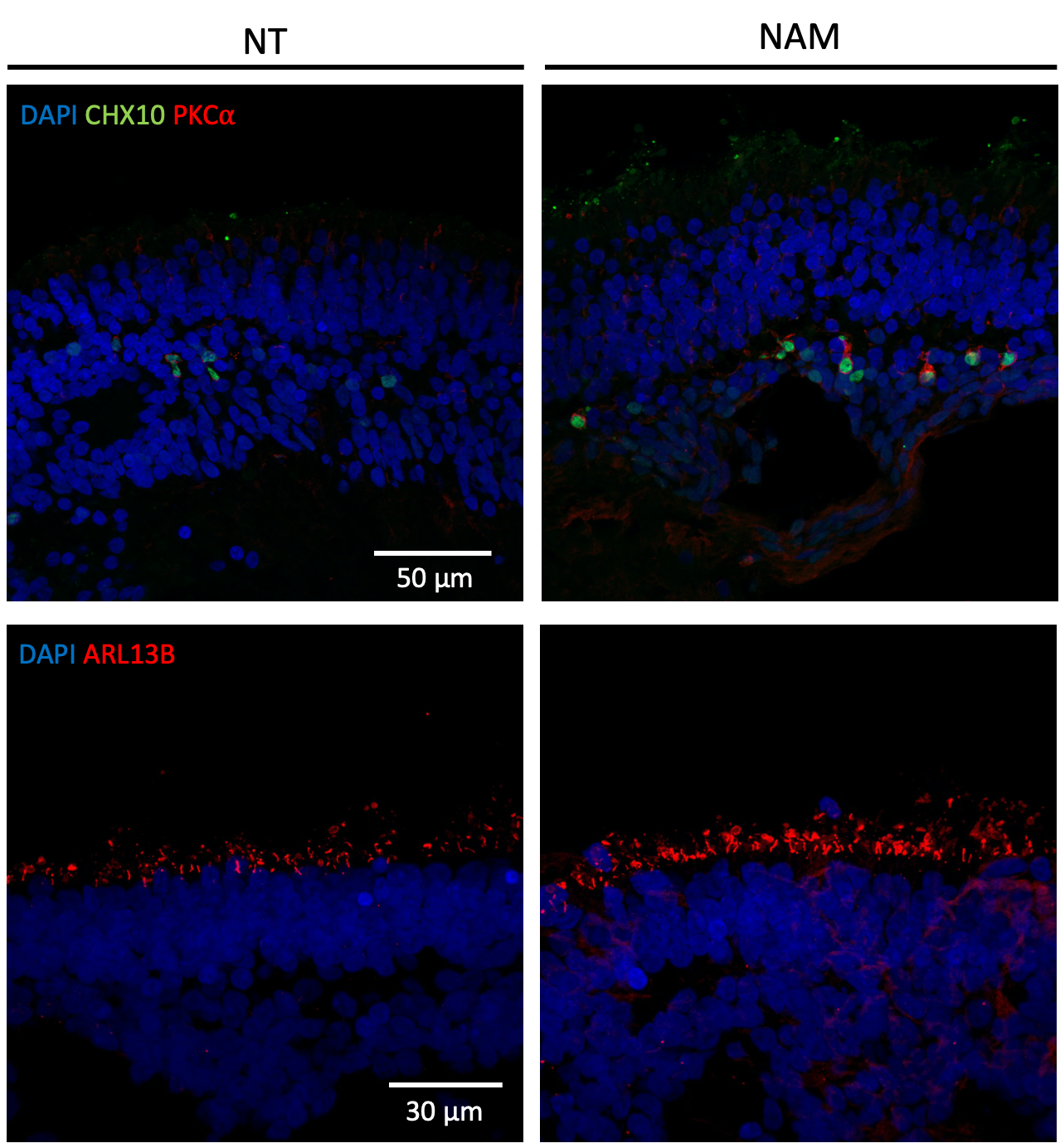
**

D180

D180

**Supplementary Figure 2.** High-resolution images of untreated (NT, left) and nicotinamide (NAM)-treated (right) retinal organoids. D180 hiPSC1-derived retinal organoids were stained using antibodies against markers for rod bipolar cells (PKCα, red; upper), retinal progenitor or bipolar cells (CHX10, green; upper), and cilia (ARL13B, red; lower). Nuclei were stained with 4′,6-diamidino-2-phenylindole (DAPI, blue). Arrowheads indicate relevant staining of each marker.


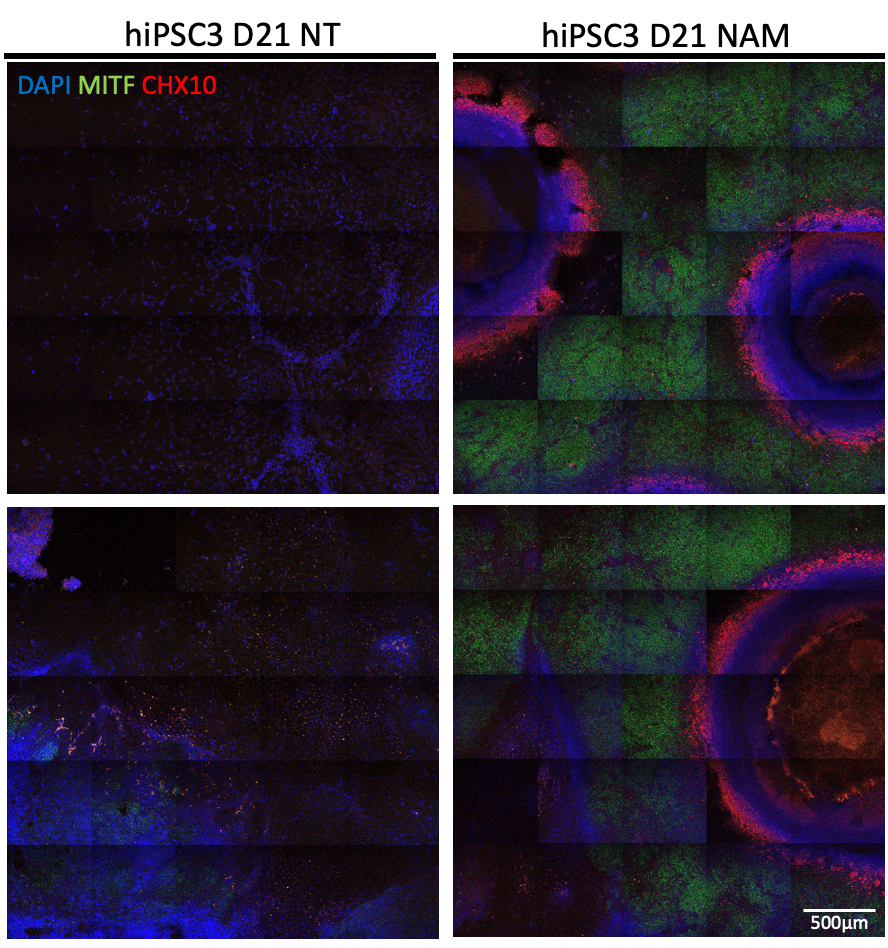


**Supplementary Figure 3.** Tile scan images of untreated (NT, left) and nicotinamide (NAM) treated (right) D21 hiPSC3 cultures. CHX10 (red) was used as a neural retinal marker and MITF (green) stained the prospective RPE region. Nuclei were stained with 4′,6-diamidino-2-phenylindole (DAPI, blue).
