# Supplementary material for: Nicotinamide Promotes Formation of Retinal Organoids From Human Pluripotent Stem Cells via Enhanced Neural Cell Fate Commitment
Source: Front Cell Neurosci. 2022 Jun 17;16:878351. doi: 10.3389/fncel.2022.878351 (PMC9247291; doi:10.3389/fncel.2022.878351)
Supplement: Supplementary file 2 [file Data_Sheet_2.doc]

**Supplemental Table 1.** Cell line information.

| **Cell line** | **Lab ID** | **Origin** | **Sex** |
| --- | --- | --- | --- |
| hiPSC1 | 8E | Control individual | Female |
| hiPSC2 | 901A | Control individual | Male |
| hiPSC3 | 804B | Control individual | Male |
| hiPSC4 | 806A | *NPHP5*-LCA patient | Female |
| hiPSC5 | 902D | *USH1C*-Usher patient | Female |
| hiPSC6 | 2F | *CEP290*-LCA patient | Female |
| hiPSC7 | 1B | *CEP290*-LCA patient | Male |
| hESC1 (H9) | H9 CRX:GFP | Control | Female |

**Supplemental Table 2.** Antibody information for immunostaining (IHC) and immunoblotting (IB)

| **Target** | **Source** | **Catalog Number** | **Host** | **Dilution** |
| --- | --- | --- | --- | --- |
| CHX10 | Abcam | ab16142 | Sheep | 1:200-1:500 (IHC); 1:1000 (IB) |
| BRN3A | Millipore Sigma | MAB1585 | Mouse | 1:200 |
| CALB | Calbiochem | PC253L | Rabbit | 1:500 |
| RHO | A gift from Dr. Robert Molday, University of British Columbia, Canada |  | Mouse | 1:500 |
| OPN1SW (N-20) | Santa Cruz | sc-14363 | Goat | 1:150-1:200 |
| PKCα | Millipore Sigma | P4334 | Rabbit | 1:500-1:1000 |
| RCVRN | Chemicon | AB5585 | Rabbit | 1:500 |
| OPN1L/MW | Millipore Sigma | AB5405 | Rabbit | 1:200-1:500 |
| PAX6 | Developmental Studies Hybridoma Bank | PAX6 | Mouse | 1:500 |
| SOX1 | R&D Systems | AF3369 | Rabbit | 1:1000 |
| ARL13B | Proteintech | 17711-1-AP | Rabbit | 1:1000 |
| MITF | Abcam | ab80651 | Mouse | 1:500 |
| β-Actin | Millipore Sigma | A5316 | Mouse | 1:1000 (IB) |
| p-SMAD1/5/9 | Cell Signaling | 13820S | Rabbit | 1:1000 (IB) |
